# Responses of Soil Microbial Biomass and Enzyme Activities to Tillage and Fertilization Systems in Soybean (*Glycine max* L.) Production

**DOI:** 10.3389/fpls.2016.01730

**Published:** 2016-11-21

**Authors:** Gholamreza Heidari, Khosro Mohammadi, Yousef Sohrabi

**Affiliations:** ^1^Department of Agronomy and Plant Breeding, University of KurdistanSanandaj, Iran; ^2^Department of Agronomy, Sanandaj Branch, Islamic Azad UniversitySanandaj, Iran

**Keywords:** compost, dehydrogenase, microbial biomass, phosphatase, urease, tillage

## Abstract

Tillage operation and fertilizer type play important roles in soil properties as far as soil microbial condition is concerned. Information regarding the simultaneous evaluation of the effect of long-term tillage and fertilization on the soil microbial traits of soybean farms is not available. Accordingly, it was hypothesized that, the microbial biomass and enzyme activity, more often than not, respond quickly to changes in soil tillage and fertilization. Therefore, the experiments were aimed at analyzing the responses of soil microbial traits to tillage and fertilization in a soybean field in Kurdistan University, Iran. The field soil is categorized into coarse Loamy, mixed, superactive, calcareous, and mesic Typic Xerorthents. The experiments were arranged in split plot, based on randomized complete block design with three replications. Main plots consisted of long-term (since 2002) tillage systems including conventional tillage (CT), minimum tillage (MT), and no-tillage (NT). Eight fertilization methods were employed in the sub-plots, including (F1): farmyard manure (FYM); (F2): compost; (F3): chemical fertilizers; (F4): FYM + compost; (F5): FYM + chemical fertilizers; (F6): compost + chemical fertilizers; (F7): FYM + compost + chemical fertilizers and (F8): Control (without fertilizer). The highest microbial biomass carbon (385.1 μg) was observed in NT-F4 treatment. The NT treatment comparatively recorded higher values of acid phosphatase (189.1 μg PNP g^−1^ h^−1^), alkaline phosphatase (2879.6 μg PNP g^−1^ h^−1^) and dehydrogenase activity (68.1 μg PNP g^−1^ h^−1^). The soil treated with a mixture of compost and FYM inputs had the maximum urease activity of all tillage treatments. Organically manured treatment (F4) showed more activity in dehydrogenase (85.7 μg PNP g^−1^ h^−1^), acid phosphatase (199.1 μg PNP g^−1^ h^−1^), and alkaline phosphatase (3183.6 μg PNP g^−1^ h^−1^) compared to those treated with chemical fertilizers. In NT-F4 treatment, using on-farm inputs is most suitable for sustainable management and improvement in soil biological activities in soybean cultivation. We concluded that applying organic manures and employing reduced tillage systems increased soil microbial biomass and enzyme activities.

## Introduction

Tillage operations, which use moldboard plowing and disking to prepare soil for sowing, can decrease soil microbial activity and organic matter (Gupta and Germida, 1988; Mohammadi et al., 2012). Continuous use of conventional tillage (CT) system, changes the physical, chemical, and biological properties of the soil (Lupwayi et al., 2012), hence the need is seen for alternative systems that reduce soil degradation and improve agricultural sustainability (Balota et al., 2004). Using minimum tillage practices for instance, can improve such physical, chemical and biological properties as macro-pore structure, aggregate stability, nutrients availability, and the diversity of microbial populations while reducing soil disturbance. Improving soil structure enhances root growth, which may in turn help the crop explore a larger volume of soil and use nutrients and water more efficiently (Mitchell et al., 2003). McAndrews et al. (2006) reported 6–13% higher soybean yield using chisel tillage with residual swine manure or compost application to land than that which only received one amendment application or a non-amended control. Soybean is an important oilseed crop in many countries around the world and its cultivation is rapidly increasing in Iran. Type and amount of fertilizers exert a great impact on soil quality (Dong et al., 2012). Organic amendments are often applied to increase soil fertility, crop quality, or both (Edmeades, 2003; Mohammadi et al., 2011, 2013). Furthermore, organic manure enhances the environmental sustainability of agricultural systems by increasing the organic matter of the soil and decreasing chemical inputs (Mohamed and Abdu, 2004). In addition to providing necessary nutrients for crops and improving soil physico-chemical properties, organic fertilizer is able to enhance soil microbial activity of soil, such as improving activity of soil enzymes and increasing soil microbial biomass (Sun et al., 2003; Lv et al., 2005; Mao et al., 2008). Co-application of organic manure and chemical fertilizers is a significant approach to maintain and improve soil fertility, and increase fertilizer use efficiency (Ming-gang et al., 2008).

Microorganisms play a crucial part in soil nutrient cycling, maintenance of soil structure, degradation of agrochemicals and pollutants, and plant pest control (Stockdale and Brookes, 2006), hence it has often been indicated as an important component of soil fertility (Nogueira et al., 2006). Enzymatic activities in the soil highly affect nutrient cycling and organic matter decomposition (Pavel et al., 2004). Phosphatases, meanwhile, are involved in the transformation of organic phosphorus compounds in soil (Amador et al., 1997). Moreover, ureases are in charge of releasing inorganic N in the N-cycle (Bandick and Dick, 1999). A case study indicated that excessive cultivation decreased both microbial biomass and its activities. (Gupta and Germida, 1988). In another survey, phosphatase and urease activities were higher in no-tillage treatment compared with the conventional and minimum tillage treatments (Mohammadi et al., 2012). The objective of the present study was to determine the effects of different soil conservation management practices as no-tillage, reduced tillage, and organic fertilizers on microbiological and biochemical soil quality indicators in a commercial soybean field. We hypothesized that in soybean systems, microbial biomass content and enzyme activity are highly influenced by the availability of soil nutrients in the tillage systems and the organic manure application leads to higher enzyme activities mainly under no tillage system.

## Materials and methods

The experiments related to the study were carried out at the Research Farm of Kurdistan University, Sanandaj, Iran, an area located at a latitude of 35°15′ N, a longitude of 47°1′ E and an altitude of 1300 m above the mean sea level. This site was chosen owing to its long-term tillage history. Divided into three tillage managements in 2002, this area had already been farmed prior to our experiments. Table 1 indicates certain soil physico-chemical properties of the experimental field. The field soil was categorized into coarse Loamy, mixed, superactive, calcareous, and mesic Typic Xerorthents (Ammari, 1987). Experiments were arranged in split plot and randomized block designs with three replications for 2 years. The size of the main plots was 15 × 20 m while the space between them was three meters. They consisted of long-term tillage systems including moldboard plowing with an average depth of 30 cm + two shallow disks (conventional tillage–CT), and chisel plowing with an average depth of 15 cm + one shallow disk (minimum tillage–MT), and no-tillage (NT) where crop residues cut by the combine were chopped and spread evenly with a combine-attached chopper. Furthermore, the NT treatments were seeded with a NT seed drill and in the CT and MT treatments, soybean seeds were sown using row planter. The size of the subplots was 1.6 × 15 m while the space between them was 1 m. We employed eight strategies so as to supply the basal fertilizer requirements of soybean in the subplots: (F1): 5400 kg FYM ha^−1^ (cattle manure); (F2): 2500 kg compost ha^−1^; (F3): 90 kg triple super phosphate ha^−1^ + 60 kg Urea ha^−1^; (F4): 2700 kg FYM ha^−1^ + 1250 kg compost ha^−1^; (F5): 2700 kg FYM ha^−1^ + 45 kg triple super phosphate ha^−1^ + 30 kg Urea ha^−1^; (F6): 1250 kg compost ha^−1^ + 45 kg triple super phosphate ha^−1^ + 30 kg Urea ha^−1^; (F7): 1800 kg FYM ha^−1^ + 833 kg compost ha^−1^ + 30 kg triple super phosphate ha^−1^ + 20 kg Urea ha^−1^ and (F8) control (without fertilizer). Organic fertilizers were also analyzed for chemical and nutrient properties according to Peters et al. (2003) method. Table 2 shows chemical characteristics of organic manures applied to the soil. Compost, FYM and chemical fertilizers were added to treatments prior to sowing the soybean. For CT and MT, chemical or organic fertilizers were applied and then incorporated with tillage, while for NT treatments, the fertilizers were surface-applied. Urea fertilizer was equally applied before sowing and at the flowering of the soybean, at R_2_ (full bloom) development stage. Adopting a plant spacing of 40 and 20 cm, the soybean seeds were sown on May 10 and 12 in 2012 and 2013 growing seasons employing Williams's cultivar, which is an early maturing soybean cultivar whose growth type is indeterminate, while its growth duration is about 135 days. The field was irrigated twice with a 7–9 day interval for a better growth and establishment of the seeds. It was also irrigated at stemming and flowering along with fertilization, and at podding and grain filling. Total water applied by irrigation during the crop cycle was about 500 mm. Weeds were removed manually in all treatments.

**Table 1:** **Soil characteristics of research field**.

**Soil texture**	**Organic carbon (g kg^−1^)**	**pH**	**Ec (dS m^−1^)**	**Clay**	**Sand**	**Silt**	**P**	**K**	**Zn**	**Fe**	**Cu**
				**(%)**			**(mg kg**^**−1**^**)**				
Sandy loam	16.20	7.60	0.56	20	52	28	4.91	78.65	5.36	11.39	2.10

**Table 2:** **Chemical characteristics of organic manures applied to the soil**.

**Characteristics**	**pH**	**N**	**P**	**K**	**Fe**	**Ca**	**Mg**	**Zn**	**Cu**	**Ca**
		**(g kg**^**−1**^**)**				**(mg kg**^**−1**^**)**				
Farmyard manure	7.44	5.1	4.8	3.0	4.0	2336	1155	7	28	2336
Compost	7.20	10.9	11.9	5.4	19.1	1671	1851	42	312	1671

For soil biological analyses, soil samples were taken at about 0–30 cm depth, and at the flowering stage of the soybean growth. Then the moist soil samples were sieved through a 5-mm mesh screen and held at 4°C until microbial analysis. Soil organic carbon (SOC) was measured via spectrophotometer, employing oxidizing organic carbon with K_2_Cr_2_O_7_ (1.5 N) in an acid environment with H_2_SO_4_ (Nelson and Sommers, 1982). Microbial biomass carbon (MBC) was evaluated by chloroform fumigation-extraction method with a kEC = 0.38 (Vance et al., 1987). The enzyme activities of acid (EC 3.1.3.2) and alkaline phosphatase (EC 3.1.3.1) were measured using *p*-nitrophenyl phosphate disodium (0.115 M) as the substrate. Two milliliters of 0.5 M sodium acetate buffer (pH 5.5) were added to 0.5 g of soil using acetic acid and 0.5 mL of substrate and then incubated at 37°C for 90 min. The reaction was terminated by a cooling degree of 2°C for 15 min. Next, 0.5 mL of 0.5 M CaCl_2_, and 2 mL of 0.5 M NaOH were added, and the mixture was centrifuged at 2287 × g for 5 min. The *p*-nitrophenol was determined by spectrophotometry at 398 nm (Tabatabai and Bremner, 1969). Dehydrogenase activity was evaluated by the reduction of triphenyl tetrazolium chloride (TTC) to triphenyl formazan (TPF) as described by Serra-Wittling et al. (1995) with modifications. Briefly, moist soil (2 g) was treated with 2.5 ml of 1% TTC–Tris buffer (pH 7.6), and then incubated at 37°C in darkness for 24 h. Urease (EC 3.5.1.5) activity was measured using 0.5 M urea as substrate in 0.1 M phosphate buffer with a pH of 7 (Nannipieri et al., 1974). The NH4+-N produced by urease activity was determined using a flow injection analyzer (FIAStar, Tecator, Sweden). To account for the NH4+-N fixation by soils, NH4+-N solutions with concentrations in the range of those released by urease activity were incubated with the soils. The values related to enzyme activities were calculated based on the oven-dry (105°C) soil weight.

Using SAS (SAS Institute, 2003), the data underwent analysis of variance. The combined analysis of variance was applied to the data for 2 years and the least significant difference (LSD) was employed in the comparison of the means (*p* < 0.05). For significant interactions, slicing was used. Prior to the combined analysis of variance, Bartlett's (1947) test of homogeneity of variances and the normality test for data were performed. In addition, orthogonal contrast analysis was separately conducted in all tillage treatments as follows:

Chemical fertilizer alone (F3 treatment) vs. Other fertilized treatments (F1, F2, F4, F5, F6, F7 treatments).
Organic manure treatments (F1, F2, F4 treatments) vs. Chemical or combined fertilizers (F3, F5, F6, F7 treatments).
Compost bearing treatments (F2, F4, F6, F7 treatments) vs. Treatments without compost (F1, F3, F5 treatments).
Farmyard manure bearing treatments (F1, F4, F5, F7 treatments) vs. Treatments without farmyard manure (F2, F3, F6 treatments).

## Results

None of the interactions between the experimental factors and year were statistically significant for studied variables (Table 3). Therefore, average of data from 2 years was used for interpretation.

**Table 3:** **Combined analysis of variance over years (2012 and 2013) for soil microbial biomass and enzyme activities affected by tillage and fertilization systems**.

**Source of Variation**	**DF**	**Soil organic carbon**	**MBC**	**Acid phosphatase**	**Alkaline phosphatase**	**Dehydrogenase**	**Urease**
Year (Y)	1	ns	ns	ns	ns	ns	ns
R/Year	4	ns	ns	ns	ns	ns	ns
Tillage systems (T)	2	ns	^**^	^**^	^**^	^**^	^**^
T^*^Y	2	ns	ns	ns	ns	ns	ns
Error a	8	–	–	–	–	–	–
Fertilization methods (F)	7	ns	^**^	^**^	^**^	^**^	^**^
F^*^Y	7	ns	ns	ns	ns	ns	ns
F^*^T	14	ns	^*^	ns	ns	ns	^**^
F^**^Y	14	ns	ns	ns	ns	ns	ns
Error b	84	–	–	–	–	–	–

ns, non-significant; ^*^ and ^**^, significant at 1 and 5% probability levels, respectively.

**Figure 1:**
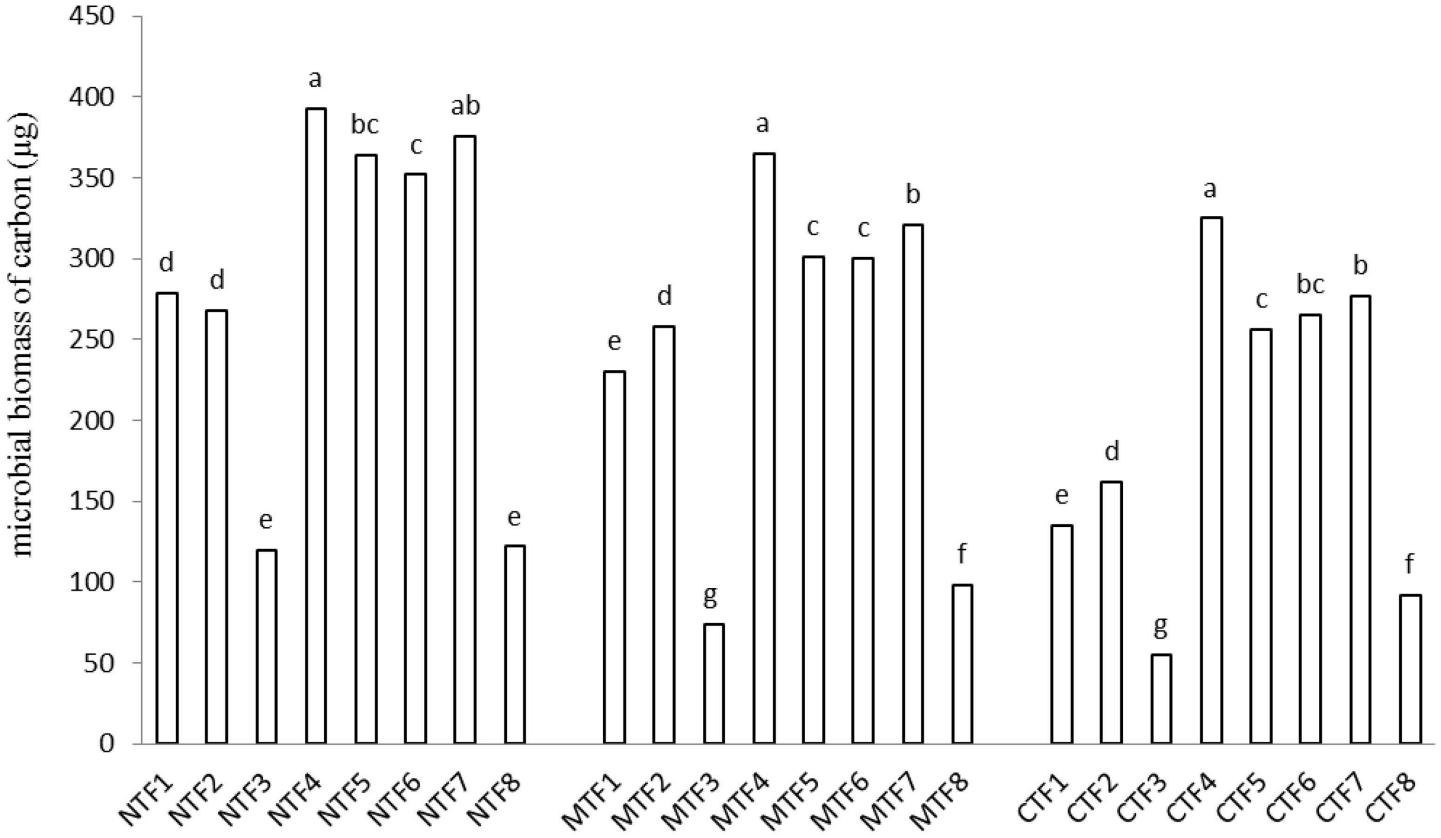
**Interaction effect of fertilization and tillage on microbial biomass of carbon**. (F1, farmyard manure; F2, compost; F3, chemical fertilizers; F4, farmyard manure + compost; F5, farmyard manure + chemical fertilizers; F6, compost + chemical fertilizers; F7, farmyard manure + compost + chemical fertilizers; F8, control & NT, No-tillage; MT, minimum tillage; CT, conventional tillage; Different letters indicate significant differences only between the different fertilization variants using LSD test at the *p* < 0.05 level). For significant interactions, slicing was used.

Tillage and fertilization treatments and the interaction of tillage × fertilization had significant effects on MBC. SOC, however, was not affected by tillage and fertilization (Table 3). Moreover, the highest MBC was observed in NT-F4 treatment. Applying organic fertilizers in the absence of tillage system resulted in an increase in the MBC, while chemical fertilizers and minimum and conventional tillage systems did not bring about any changes (Figure 1). Contrast analysis showed that in all tillage treatments, chemical fertilizers alone reduced the MBC compared with other fertilized treatments (Table 4).

**Table 4:** **Orthogonal analysis for soil organic carbon, microbial biomass, and soil enzyme activity affected by fertilization in tillage systems**.

	**Contrast**	**SOC**	**MBC**	**Dehydrogenase**	**Acid phosphatase**	**Alkaline phosphatase**	**Urease**
NT	Chemical fertilizer alone (F3)	16.30	122.09	43.70	143.51	2178.06	31.12
	Other fertilized treatments (F1, F2, F4, F5, F6, F7)	16.23	341.41	78.96	183.08	2662.17	55.16
	*P*	ns	^**^	^**^	^**^	^**^	^**^
	Organic manure treatments (F1, F2, F4)	16.35	314.50	80.76	178.66	2756.66	65.33
	Chemical or combined fertilizers (F3, F5, F6, F7)	16.20	306.75	68.80	176.50	2470.25	41.50
	*P*	ns	ns	^**^	ns	^**^	^**^
	Compost bearing treatments (F2, F4, F6, F7)	16.22	349.37	81.60	189.12	2786.50	56.75
	Without compost treatments (F1, F3, F5)	16.26	257.61	63.70	161.83	2335.10	45.05
	*P*	ns	^**^	^**^	^**^	^**^	^**^
	Farmyard manure bearing treatments (F1, F4, F5, F7)	16.25	355.62	80.41	187.87	2860.51	56.10
	Without farmyard manure treatments (F2, F3, F6)	16.23	249.33	65.30	163.50	2236.32	46.00
	*P*	ns	^**^	^**^	^**^	^**^	^**^
MT	Chemical fertilizer alone (F3)	16.15	80.12	37.70	135.12	2076.18	28.12
	Other fertilized treatments (F1, F2, F4, F5, F6, F7)	16.27	297.36	74.71	171.83	2512.33	47.33
	*P*	ns	^**^	^**^	^**^	^**^	^**^
	Organic manure treatments (F1, F2, F4)	16.38	289.33	77.26	169.33	2615.67	53.05
	Chemical or combined fertilizers (F3, F5, F6, F7)	16.22	250.51	63.55	164.50	2325.75	38.25
	*P*	ns	^**^	^**^	ns	^**^	^**^
	Compost bearing treatments (F2, F4, F6, F7)	16.27	310.50	77.72	179.75	2675.33	47.62
	Without compost treatments (F1, F3, F5)	16.23	209.33	58.36	149.00	2147.33	40.50
	*P*	ns	^**^	^**^	^**^	^**^	^**^
	Farmyard manure bearing treatments (F1, F4, F5, F7)	16.28	307.25	76.02	172.50	2667.75	51.25
	Without farmyard manure treatments (F2, F3, F6)	16.21	211.72	60.63	158.66	2157.44	36.66
	*P*	ns	^**^	^**^	ns	^**^	^**^
CT	Chemical fertilizer alone (F3)	16.20	40.00	31.70	126.50	1974.20	19.00
	Other fertilized treatments (F1, F2, F4, F5, F6, F7)	16.24	235.08	67.96	158.08	2400.00	40.66
	*P*	ns	^**^	^**^	^**^	^**^	^**^
	Organic manure treatments (F1, F2, F4)	16.33	204.16	68.76	155.01	2474.66	43.06
	Chemical or combined fertilizers (F3, F5, F6, F7)	16.16	209.50	58.30	152.50	2237.50	33.50
	*P*	^**^	ns	^**^	ns	^**^	^**^
	Compost bearing treatments (F2, F4, F6, F7)	16.24	255.62	73.85	170.37	2567.50	41.87
	Without compost treatments (F1, F3, F5)	16.23	142.66	48.03	131.16	2034.62	31.83
	*P*	ns	^**^	^**^	^**^	^**^	^**^
	Farmyard manure bearing treatments (F1, F4, F5, F7)	16.23	246.87	67.90	153.37	2531.25	41.50
	Without farmyard manure treatments (F2, F3, F6)	16.23	154.34	55.96	153.83	2083.12	32.33
	*P*	ns	^**^	^**^	ns	^**^	^**^

F1, farmyard manure; F2, compost; F3, chemical fertilizers; F4, farmyard manure + compost; F5, farmyard manure + chemical fertilizers; F6, compost + chemical fertilizers; F7, farmyard manure + compost + chemical fertilizers; F8, control & NT, No-tillage; MT, minimum tillage; CT, conventional tillage. ns ,^*^ and ^**^: not significant, significant at p < 0.05 and p < 0.01 of probability, respectively.

Organically manured treatment (F4) led to higher acid and alkaline phosphatase activities compared to treatments with chemical fertilizers. The highest acid and alkaline phosphatase activities were found in F4 treatment. There was no significant difference, however, between F4 and F7 as far as alkaline phosphatase activity is concerned (Table 5). The orthogonal contrasts revealed a significant difference between the organic manure treatments and chemical or combined fertilizers in all tillage treatments (Table 4). The highest acid and alkaline phosphatase activities were observed in the NT treatment (189.1 and 2879.6 μg PNP g^−1^ h^−1^) while the lowest were observed in the CT treatment (125.9 and 1989.6 μg PNP g^−1^ h^−1^) (Table 5).

**Table 5:** **Effect of fertilization methods and tillage systems on soil organic carbon and soil enzyme activity**.

**Fertilization**	**Soil organic carbon (g kg^−1^)**	**Acid phosphatase (μg PNP g^−1^ h^−1^)**	**Alkaline phosphatase (μg PNP g^−1^ h^−1^)**	**Dehydrogenase (μg PNP g^−1^ h^−1^)**
FYM (F1)	16.261 a	136.611 d	2347.912 b	68.498 b
Compost (F2)	16.312 a	168.392 c	2317.288 b	76.388 ab
Chemical fertilizer (F3)	16.198 a	135.901 d	2076.918 c	37.701 c
FYM + Compost (F4)	16.401 a	199.162 a	3183.612 a	85.712 a
FYM + Chemical (F5)	16.308 a	171.694 bc	2094.191 c	67.692 b
Compost + Chemical (F6)	16.289 a	173.215 bc	2086.506 c	67.925 b
FYM + Compost + Chemical (F7)	16.279 a	179.424 b	3122.299 a	81.801 a
Control (F8)	16.198 a	104.926 e	2049.105 c	34.612 c
**TILLAGE**				
No Tillage (NT)	16.322 a	189.172 a	2879.663 a	68.159 a
Minimum Tillage (MT)	16.286 a	159.857 b	2358.958 b	62.886 b
Conventional Tillage (CT)	16.217 a	125.976 c	1989.693 c	63.796 b

*Mean values in each column with the same letter(s) are not significantly different using LSD tests at 5% of probability. (FYM: farmyard manure)*.

**Figure 2:**
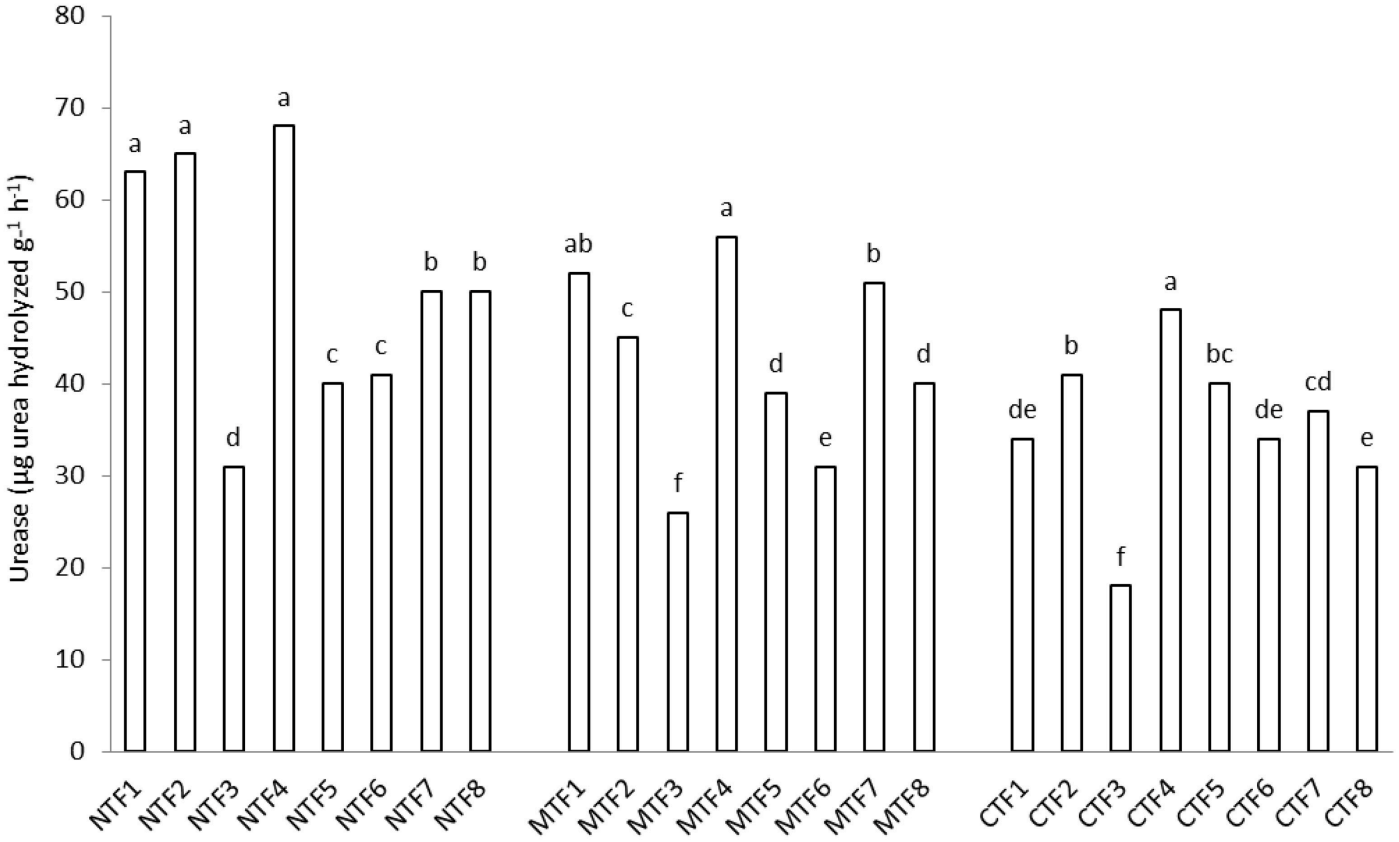
**Interaction effect of fertilization and tillage on urease activity**. (F1, farmyard manure; F2, compost; F3, chemical fertilizers; F4, farmyard manure + compost; F5, farmyard manure + chemical fertilizers; F6, compost + chemical fertilizers; F7, farmyard manure + compost + chemical fertilizers; F8, control & NT, No-tillage; MT, minimum tillage; CT, conventional tillage; Different letters indicate significant differences only between the different fertilization variants using LSD test at the *p* < 0.05 level). For significant interactions, slicing was used.

Dehydrogenase activity was significantly higher in soils receiving organic fertilizers (F4) than those under chemical fertilizer treatment. However, there was no significant difference among F4 and F2 and F7 treatments as far as dehydrogenase activity is concerned (Table 5). Dehydrogenase activity was at its lowest in control treatment (Table 4). Contrast analysis showed that in all tillage treatments, organic manure treatments increased the dehydrogenase activity unlike other fertilized treatments (Table 4). Dehydrogenase activity tended to be higher in the NT (68.1 μg PNP g^−1^ h^−1^) compared with MT (62.8 μg PNP g^−1^ h^−1^) and CT (63.7 μg PNP g^−1^ h^−1^) treatments (Table 5). Furthermore, urease activity was significantly affected by the interaction of fertilization × tillage (Figure 2). Applying chemical fertilizers to CT treatment (CT-F3) significantly decreased urease activity, whereas adding organic manure to NT treatment (NT-F4) resulted in an increase in its activity (Figure 2). With compost bearing treatments, the urease activity was increased by 41.6, 17.5, and 31.5% in NT, MT, and CT treatments respectively. Moreover, compared to treatments without FYM, in FYM bearing treatments, the urease activity was increased by 21.9, 17.5, and 28.3% in the NT, MT, and CT treatments respectively (Table 4).

## Discussion

Organic manure treatments had more MBC than those receiving chemical fertilizers, which could be the result of the increase in organic N, carbohydrate and water soluble C (Mohammadi et al., 2012). Contrast analysis results indicated that in all tillage treatments, soils receiving compost and FYM bearing treatments had larger MBC pools than those without compost and FYM (Table 4). The increase in MBC after applying organic manures indicates that soil organic carbon provided by organic manures may have been consumed as energy source by microorganisms (Fauci and Dick, 1994; Sarathchandra et al., 2001; Min et al., 2003). In an experiment conducted by Lupwayi et al. (2012) at two barley farms in western Canada, in a Black Chernozem, the MBC increased through the application of up to 60 kg ha^−1^ chemical nitrogen fertilizer and decreased employing more than 120 kg ha^−1^. In China, results of a certain study revealed that chemical nitrogen fertilizers of more than 160 kg ha^−1^ had negative effects on soil microbial biomass in grassland (Zhang et al., 2008). The results of the present research indicated that unlike CT treatment, the NT treatment increased MBC in all fertilization levels (Figure 1). Higher surface MBC concentrations resulting from NT and MT treatments may have occurred owing to the accumulation of crop residues and organic manure in soil. Findings of Prakash et al., 2007 showed that the addition of FYM along with NPK fertilizers increased SOC by 37.8%, over NPK-alone treatment. Furthermore, applying FYM along with N or NPK resulted in significantly higher MBC and populations of microorganisms in comparison with chemically fertilized treatments. It should be noted that our study was not long enough for us to determine whether or not chemical fertilizers decreased SOC, hence this is a fact that our results cannot be explained through the above mentioned mechanism. Another cause of the reduction in MBC was the decrease in soil pH which is the most important chemical factor controlling soil microbial communities (Wallenstein et al., 2006).

The positive effects of conservation tillage on the accumulation of soil carbon have been proven by Spedding et al. (2004). While fertilization and tillage had no significant effect on SOC, in NT soils SOC increased with the increase in organic manure (Table 5) suggesting the beneficial co-effects of no-tillage and organic manures. The increase in soil organic carbon depends on many factors such as the no-tillage years (Zhang and Song, 2003), soil depth (Treonis et al., 2010), and soil type (Helgason et al., 2009). Zhang and Song (2003) have claimed that SOC, compared to MBC, needs a longer period to respond to no-tillage and residue amendment. Because of its quicker turnover rate, the changes in MBC are more apparent than soil organic carbon over short periods. Moreover, SOC needs more time to respond to NT and organic matters compared with MBC (Zhang and Song, 2003).

The phosphatase, dehydrogenase and urease enzymes studied in our experiment have a principal role in nutrient cycles of soil. Measuring the activities of the aforementioned enzymes may help assess the changes in organic carbon. The contrast analysis revealed that in all tillage systems, chemical fertilizers, unlike the rest of the fertilizers, decreased the enzymes activity (Table 4). A possible reason might be that organic matters could increase microbial activity in the soil. Acid and alkaline phosphatase activity in the soil significantly depended on the type of organic manures and whether or not chemical fertilizers were employed. Higher acid and alkaline phosphatase activities of soil treated with organic manures could be related to their microbial biomass production (Figure 1). Increased phosphatase activity could change the insoluble phosphate into free ions later taken up by crops. Tarafdar and Marschner (1994) demonstrated that plants can utilize insoluble phosphate compounds from the soil by increasing the phosphatase activity. Chemical fertilizers suppressed the acid and alkaline phosphatase activity which can be explained through considering the fact that phosphatase synthesis is inhibited by available phosphorus (Wang et al., 2008). Additionally, applying more compost (90–270 t ha^−1^), linearly increased the phosphatase activities (Giusquiani et al., 1994).

Dehydrogenase activity exists as an essential part of soil microbial life (Wlodarczyk et al., 2002). It is worth noting that organic fertilizers are more favorable to the overall biological activity of the soil compared with mineral fertilizers which can exert a negative effect on the activity of the enzyme. In the present research, NT treatment brought about a significantly higher soil dehydrogenase activity in comparison with CT and MT treatments, a result consistent with the findings of Doran (1980).

Application of chemical fertilizer decreased urease activity (Tables 4, 5). Urea fertilizers and the reaction products of urease have ammonium causing microbial induction of urease activity to decrease in chemical fertilizer treatments, which is in line with the findings reported by Martens et al. (1992). Furthermore, our result is in consistent with the findings of Saha et al. (2008) who indicated that organic manure had a positive effect on urease activity. Using FYM alone increased urease activity in NT and MT treatments but in CT treatment such an increase was observed only when FYM was employed along with compost (Table 4). Overmuch tillage and crop residue burying in the CT resulted in a significant decrease in soil organic matter (Zibilske et al., 2002). However, soil surface residues accumulated as CT can increase soil organic matter and microbial biomass (Salinas-Garcia et al., 2002). Therefore, it seems reasonable that higher amounts of organic matter could provide better conditions for the accumulation of enzymes in the soil. Moreover, the higher the urease activities were in NT the higher was the productivity. It should be mentioned that the 10 years various soil tillage practices had caused significant differences in soil chemical and physical properties. The generally higher urease activities in NT mainly resulted from the larger water availability in this treatment rather than the better soil fertilities. The NT maintains residues near the soil surface, minimizes soil erosion and increases water infiltration (Jin et al., 2009).

In addition to soil microbial traits, conservation tillage systems (NT and MT) have great potentials for stabilizing production in semiarid conditions and have higher grain yields than the CT (Moreno et al., 2011), a fact also consistent with our results (data not shown). In addition, organic manures could increase the crop yield, due to the improvement of soil physical structure and nutrient accessibility. Consequently, greater enzyme activity and microbial biomass were the results of the fact that more root exudates were released into the soil by the plants (Zhong et al., 2010).

## Conclusion

The present study provides information on soil microbial biomass parameters as influenced by tillage, organic and inorganic fertilization in soybean production conditions. We concluded that on a short-term basis, MBC and soil enzyme activities are dependent on whether organic manures or chemical fertilizers are applied. Soil microbial biomass and enzymatic properties were also closely related with the carbon inputs. These differences were most pronounced between no-tillage and conventional and reduced tillage. In NT-F4 treatment, using on-farm inputs is most suitable for sustainable management and improvement in soil biological activities in soybean cultivation where a low input system can be carried out. Our results support the hypothesis that the microbial biomass and enzyme activity, more often than not, respond quickly to changes in soil tillage and fertilization. The NT system, which largely increases the surface of crop residues, is the most effective method for improving soil microbial biomass of carbon and enzymes activities in a relatively short term and for sustaining agricultural ecosystems.

## Author contributions

All authors listed, have made substantial, direct and intellectual contribution to the work, and approved it for publication.

### Conflict of interest statement

The authors declare that the research was conducted in the absence of any commercial or financial relationships that could be construed as a potential conflict of interest.

## Abbreviations

CT: conventional tillage
MT: minimum tillage
NT: no-tillage
FYM: farmyard manure
SOM: soil organic matter
MBC: microbial biomass carbon.
